# Selective Motor Entropy Modulation and Targeted Augmentation for the Identification of Parkinsonian Gait Patterns Using Multimodal Gait Analysis

**DOI:** 10.3390/life16020193

**Published:** 2026-01-23

**Authors:** Yacine Benyoucef, Jouhayna Harmouch, Borhan Asadi, Islem Melliti, Antonio del Mastro, Pablo Herrero, Alberto Carcasona-Otal, Diego Lapuente-Hernández

**Affiliations:** 1 Spacemedex, 930 Route des Dolines, 06560 Valbonne, France; 2 Department of Physiatry and Nursing, Faculty of Health Sciences, University of Zaragoza, 50009 Zaragoza, Spain; basadi@iisaragon.es (B.A.); pherrero@unizar.es (P.H.); d.lapuente@unizar.es (D.L.-H.); 3 iHealthy Research Group, IIS Aragon, 50009 Zaragoza, Spain; 4 National Institute of Technology and Applied Sciences, Centre Urbain Nord BP 676, Tunis 1080, Tunisia; mila.melliti@gmail.com; 5 Tunis National School of Engineering, Faculty of engineering, BP 37, Le Belvédère, Tunis 1002, Tunisia; 6 Aldebran/Mars Planet Technologies, Via Dalmine 10/A, 24035 Curno, BG, Italy; pub@marsplanet.org

**Keywords:** Parkinsonian gait, gait analysis, selective data augmentation, motor variability, motor entropy, time-series classification, wearable sensors

## Abstract

Background/Objectives: Parkinsonian gait is characterized by impaired motor adaptability, altered temporal organization, and reduced movement variability. While data augmentation is commonly used to mitigate class imbalance in gait-based machine learning models, conventional strategies often ignore physiological differences between healthy and pathological movements, potentially distorting meaningful motor dynamics. This study explores whether preserving healthy motor variability while selectively augmenting pathological gait signals can improve the robustness and physiological coherence of gait pattern classification models. Methods: Eight patients with Parkinsonian gait patterns and forty-eight healthy participants performed walking tasks on the Motigravity platform under hypogravity conditions. Full-body kinematic data were acquired using wearable inertial sensors. A selective augmentation strategy based on smooth time-warping was applied exclusively to pathological gait segments (×5, σ = 0.2), while healthy gait signals were left unaltered to preserve natural motor variability. Model performance was evaluated using a hybrid convolutional neural network–long short-term memory (CNN–LSTM) architecture across multiple augmentation configurations. Results: Selective augmentation of pathological gait signals achieved the highest classification performance (94.1% accuracy, AUC = 0.97), with balanced sensitivity (93.8%) and specificity (94.3%). Performance decreased when augmentation exceeded an optimal range of variability, suggesting that beneficial augmentation is constrained by physiologically plausible temporal dynamics. Conclusions: These findings demonstrate that physiology-informed, selective data augmentation can improve gait pattern classification under constrained data conditions. Rather than supporting disease-specific diagnosis, this proof-of-concept study highlights the importance of respecting intrinsic differences in motor variability when designing augmentation strategies for clinical gait analysis. Future studies incorporating disease-control cohorts and subject-independent validation are required to assess specificity and clinical generalizability.

## 1. Introduction

Gait analysis has emerged as a valuable tool for studying neurodegenerative disorders and motor dysfunction, including Parkinsonian syndromes, in which progressive alterations in neuromuscular control lead to subtle yet clinically meaningful changes in walking dynamics [1,2]. Beyond overt motor symptoms, Parkinsonian gait is commonly associated with impaired motor adaptability and altered movement variability, reflecting disrupted physiological regulation of locomotion.

In parallel, machine learning approaches have increasingly been applied to gait analysis, often using data augmentation to address class imbalance and limited datasets. Recent work has also explored optimization-driven approaches for gait pattern recognition, including metaheuristic-based frameworks designed to enhance feature learning and classification performance in sports and clinical gait analysis [3]. However, in multimodal biomedical contexts, the indiscriminate augmentation of both healthy and pathological data may introduce unrealistic inter-class variance and distort physiologically meaningful movement patterns, potentially limiting model generalizability [4,5,6]. Two persistent challenges, therefore, remain in pathological movement classification: capturing the temporal complexity of gait signals and achieving robust generalization when cohort sizes are small. Importantly, many current approaches overlook global dynamical properties of movement, such as motor variability and complexity, despite their established relevance as indicators of underlying physiological state [7,8].

Our previous work established the methodological foundation for the present study by combining wearable inertial measurement units (IMUs) with a hypogravity-based robotic rehabilitation platform to capture high-resolution gait kinematics in both healthy individuals and individuals presenting Parkinsonian gait patterns [9,10]. Using a hybrid convolutional neural network (CNN) and long short-term memory (LSTM) architecture, we demonstrated that spatiotemporal gait signals could be effectively modeled and classified with high internal performance. Although these pilot studies were limited by small cohorts and controlled experimental conditions, they revealed consistent discriminative patterns and suggested that variability-related descriptors may provide informative signatures of neuromotor integrity [11,12,13].

In our previous study [10], augmentation was applied to healthy control recordings to compensate for limited data availability and support comparative model training. In the present work, we refine this strategy by introducing a physiology-informed rationale to preserve healthy gait variability while selectively augmenting pathological gait signals. This approach is motivated by the observation that healthy locomotion typically exhibits richer adaptive variability, whereas Parkinsonian gait may present reduced or abnormally structured variability in timing and motor control [14,15,16].

Building on these findings, this pilot study revisits data augmentation through the conceptual lens of motor entropy, understood here as the intrinsic variability and complexity that characterize adaptive movement [17]. Rather than treating augmentation as a purely technical operation, we hypothesize that preserving the natural variability of healthy gait while selectively modulating temporal dynamics of pathological gait signals more faithfully reflects the physiological distinctions between classes and may improve classification behavior under constrained data conditions. Within this framework, higher motor entropy is associated with adaptive neuromuscular control, whereas reduced entropy reflects diminished complexity and reduced flexibility in Parkinsonian gait patterns [18]. Importantly, this study is positioned as a proof-of-concept methodological investigation and does not aim to establish disease-specific diagnostic claims. Instead, it seeks to inform the design of more physiologically coherent augmentation strategies for gait-based time-series analysis, as a foundation for future work incorporating larger cohorts, disease-control populations, and subject-independent validation.

## 2. Materials and Methods

### 2.1. Study Design

This investigation followed a cross-sectional observational approach, utilizing pre-existing data from individuals diagnosed with Parkinson’s disease (PD) as well as healthy control participants. The patient datasets originated from a previous clinical trial conducted in 2019 at the Aragon Parkinson’s Association in Zaragoza, Spain. The original protocol received ethical clearance from the Aragon Research Ethics Committee (CEICA) under reference C.P.-C.I. PI18/386 and adhered fully to the Declaration of Helsinki and other applicable ethical standards. Prior to participation, all volunteers were provided with detailed explanations of the study’s objectives, procedures, and potential risks—such as muscle fatigue or dizziness related to the movement assessments—and written informed consent was obtained. Although the specific datasets analyzed here have not been published previously, their collection took place under the same clinical agreement and ethical approval framework as the initial study [9].

An expanded dataset, relative to our earlier work, was utilized and comprised 48 healthy subjects who performed three gait-related tasks: walking, running, and step decomposition. While these additional tasks were collected to broaden the range of locomotor patterns available in the dataset for methodological development, the analyses reported in the present study focused exclusively on walking sequences to ensure both methodological and physiological comparability with the PD group, which was restricted to walking tasks. Walking was selected as it represents the most clinically relevant and consistently impaired motor activity in Parkinson’s disease. In total, the dataset included 1989 healthy CSV segments (across the three tasks) and 196 walking sequences from 8 PD subjects. Only walking segments were used for training, validation, and augmentation procedures. This study was not designed to support disease-specific diagnostic conclusions, but rather to evaluate selective augmentation strategies under constrained data conditions.

### 2.2. Participants

Recruitment and outreach were conducted through informational meetings at the Parkinson’s association, during which potential participants were briefed on the study’s aims, methodology, eligibility requirements, and potential risks. Enrollment was voluntary and open to association members.

For the PD group, inclusion criteria required a neurologist-confirmed diagnosis of idiopathic PD, the ability to walk short distances without physical assistance, and a stable clinical status allowing safe participation in the motor tasks. Exclusion criteria included severe cognitive impairment, orthopedic conditions significantly affecting gait, and the presence of other neurological disorders. These criteria were defined to ensure both participant safety and the acquisition of clinically interpretable gait data.

The healthy control group was recruited independently in 2020 from students. Inclusion criteria for controls included the absence of neurological, musculoskeletal, or balance-related disorders, no history of movement impairment, and the ability to perform walking assessments without assistance. Although the PD and control groups originated from distinct populations, all participants performed the same walking tasks under a comparable experimental protocol, enabling comparison of gait dynamics within the scope of this pilot study.

Demographic variables such as age, sex, and disease stage were not explicitly controlled for in this pilot study, as the primary objective was methodological and physiological rather than epidemiological. These variables are therefore considered potential sources of variability and are acknowledged as limitations.

### 2.3. Equipment

The Motigravity system, developed by Aldebran Srl, is a rehabilitation device originally designed to replicate spaceflight operational constraints and subsequently adapted for physical therapy and medical rehabilitation applications. It features a convex treadmill platform capable of multidirectional movement integrated within an otherwise unidirectional treadmill structure [19]. A virtual reality headset provides a fully immersive 360° visual environment, enhancing participant engagement during training sessions. Partial weightlessness is achieved through a suspension mechanism with a harness system that unloads a controlled portion of body weight, thereby reducing musculoskeletal demand and facilitating gait execution under hypogravity conditions [20].

In this investigation, healthy participants trained using the convex treadmill configuration, whereas individuals with PD performed walking tasks on the unidirectional treadmill variant. Both configurations employed comparable body-weight support mechanisms to provide comparable hypogravity conditions across groups. The use of two treadmill setups was motivated by safety and accessibility considerations rather than experimental differentiation: the convex platform is optimized for healthy or athletic populations, while the unidirectional treadmill offers greater stability and easier access for participants with PD. Nevertheless, treadmill configuration may influence gait biomechanics (e.g., balance demands and stride regulation), and this potential confounding factor is explicitly acknowledged as a limitation of the present study.

Full-body motion capture was performed using the Perception Neuron 2.0 (PN 2.0) system, a validated inertial-based tracking technology widely used in biomechanics, rehabilitation, and movement analysis research [21]. The system consists of 17 compact inertial sensors (“neurons”) positioned on key anatomical landmarks: head, upper back, lower back, shoulders (left and right), upper arms (left and right), forearms (left and right), hands (left and right), upper legs (left and right), lower legs (left and right), and feet (left and right). Each sensor integrates a tri-axial accelerometer (X, Y, Z) to measure linear acceleration, a tri-axial gyroscope (X, Y, Z) to capture angular velocity, and a tri-axial magnetometer (X, Y, Z) to estimate orientation relative to the Earth’s magnetic field, with known sensitivity to environmental interference.

Data acquisition and real-time visualization were performed using Axis Neuron software(version 3.8.42.8591), which also supports exporting kinematic data in numerical formats and BioVision Hierarchy (BVH) files for subsequent processing and analysis.

### 2.4. Assessment and Data Processing Methods

The methodological framework builds on our previous work [10], with a key refinement that integrates motor entropy as a guiding physiological principle for data augmentation. Whereas augmentation in our earlier study was applied exclusively to healthy control recordings to address control scarcity, the present work explicitly preserves healthy motor variability and selectively augments pathological gait patterns to reflect disease-related alterations in movement dynamics. Here, motor entropy is used as a conceptual descriptor of movement variability and complexity rather than a formally computed entropy metric.

Gait recordings were obtained using the Perception Neuron 2.0 inertial motion capture system (Noitom, Beijing, China), complemented by synchronized video recordings for visual verification [22]. Raw inertial signals were first inspected to identify clinically relevant intervals based on signal integrity and video alignment. Selected segments were resampled to 25 Hz, preprocessed with a Savitzky–Golay filter to smooth local fluctuations while preserving gait events, and then low-pass Butterworth-filtered to attenuate high-frequency noise and improve signal stability. All signals were subsequently normalized to zero mean and unit variance to ensure comparability across sensors.

Segmentation was performed using a fixed-length, non-overlapping sliding window of 5 s (125 samples). Each segment inherited the label of the originating subject (Healthy or Parkinson’s disease), and incomplete or corrupted segments were excluded. To systematically evaluate the impact of augmentation strategies, four experimental conditions were compared: (i) no Augmentation, using only original gait segments; (ii) healthy-only augmentation, in which synthetic variability was introduced exclusively into healthy control segments; (iii) full-class augmentation, where both healthy and pathological segments were augmented; and (iv) selective PD-only augmentation, in which healthy gait segments were left unaltered to preserve physiological variability, while controlled time-warping was applied exclusively to Parkinsonian gait segments.

Selective PD-only augmentation was implemented by generating five synthetic variants per pathological segment using smooth time-warping with Gaussian perturbations (σ = 0.2). This transformation was chosen to introduce physiologically plausible temporal variability without altering the underlying biomechanical structure of the gait signal, thereby expanding pathological intra-class variability while respecting known constraints of motor timing regulation.

Feature selection was performed using a Random Forest classifier to rank input features by importance (sensor-channel components), followed by iterative subset testing to retain the most informative channels. The final input representation consisted of fixed-length segments of 125 time steps × 64 retained channels, which were processed using a hybrid convolutional neural network–long short-term memory (CNN–LSTM) architecture designed to capture local spatial patterns and longer-range temporal dependencies without artificially imposing variability. Dropout and batch normalization were applied to reduce overfitting, and the model was trained using the Adam optimizer (learning rate = 0.001, batch size = 32). Performance should therefore be interpreted as an internal comparative evaluation under constrained data conditions rather than a subject-independent generalization.

### 2.5. Data Collection

Prior to gait data acquisition, each participant underwent a standardized posture calibration protocol to ensure accurate alignment of the inertial measurement units (IMUs) integrated into the Perception Neuron 2.0 motion capture system. Calibration consisted of four static reference poses: Steady Pose (upright standing with arms relaxed), A-Pose (arms abducted 30–45° with palms facing downward), T-Pose (arms extended horizontally at 90°), and S-Pose (knees slightly flexed with arms projected forward at approximately 45°). Each pose was maintained for several seconds and recorded using Axis Neuron software, minimizing initialization bias and ensuring a consistent biomechanical reference frame across participants. This calibration step was essential to preserve the integrity of temporal and dynamical gait features subsequently analyzed.

Following calibration, participants performed walking tasks on the Motigravity platform under controlled hypogravity conditions. Walking was conducted at a self-selected, comfortable pace, without externally imposed constraints on stride length, cadence, or arm movement, to capture natural inter-individual variability within the experimental setup. Hypogravity support settings were kept consistent within each group and across trials to maintain stable unloading conditions during data acquisition. For consistency with the Parkinson’s disease (PD) group, which was restricted to walking trials, only walking sequences from healthy participants were retained for the main comparative analyses.

Each recording session comprised multiple walking trials, with inertial data acquisition synchronized to video recordings, enabling both quantitative kinematic analysis and qualitative verification. Total recording duration ranged from approximately 3 to 10 min per participant, depending on walking speed, endurance, and the number of gait cycles completed. This variability was intentional and addressed analytically through fixed-length segmentation, allowing comparable temporal analysis across participants while capturing spontaneous, unconstrained gait dynamics.

For safety, participants with PD walked under continuous supervision, with support from the Motigravity rehabilitation harness system, thereby minimizing fall risk. Healthy control participants completed the task with minimal assistance.

### 2.6. Analysis

Model performance was evaluated by comparing training and validation metrics across the different augmentation strategies described above. For each experimental configuration, training and validation losses and training and validation accuracies were recorded at each epoch. Loss curves were generated to visualize the evolution of categorical cross-entropy during training, while accuracy curves tracked the proportion of correctly classified samples over time [23]. All reported metrics were computed at the segment level and interpreted as internal comparative indicators within the present dataset.

Confusion matrices were computed for each configuration to quantify true positives, true negatives, false positives, and false negatives. Receiver Operating Characteristic (ROC) curves were generated by plotting the true positive rate against the false positive rate across multiple decision thresholds, and the area under the ROC curve (AUC) was calculated to summarize overall discriminative performance. ROC curves and AUC values were computed on the validation set for each configuration. These metrics were used to compare the effects of different augmentation strategies rather than to provide population-level estimates.

All analyses were performed using Python 3.8. Model training histories were obtained with TensorFlow, while confusion matrices and ROC analyses were generated with scikit-learn. All figures were generated with Matplotlib (version 3.0.3) using consistent axis scaling and annotations to ensure consistent visualization across experimental conditions.

## 3. Results

Across all evaluated configurations, models trained with selective augmentation of Parkinsonian gait segments from the PD cohort consistently outperformed other augmentation strategies, including full-class augmentation, healthy-only augmentation, and no augmentation (Table 1). While all strategies yielded relatively high performance levels, differences emerged in classification accuracy and discriminative capability across conditions. All reported results reflect comparative evaluation at the segment level within the present dataset.

Full-class augmentation increased the size of the training dataset but introduced additional variability across both classes, leading to reduced class separability compared with selective augmentation. Healthy-only augmentation increased variability within control samples but did not substantially improve discrimination of pathological gait patterns. Models trained without augmentation achieved reasonable baseline accuracy but performed worse overall across all metrics.

By contrast, selective augmentation applied exclusively to PD cohort gait segments achieved the highest overall performance, with 94.1% accuracy and an area under the ROC curve (AUC) of 0.97. Sensitivity and specificity values were closely balanced, indicating comparable performance in identifying Parkinsonian gait patterns and correctly classifying healthy controls (Table 1).

**Figure 1:**
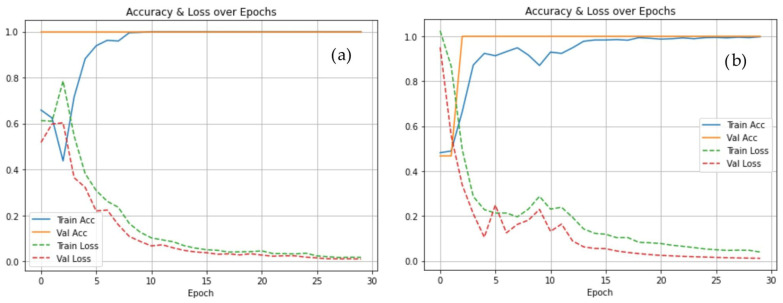
Training and validation accuracy and loss curves across epochs: (**a**) no augmentation; (**b**) selective augmentation applied to PD sequences.

Training and validation loss curves under the selective PD augmentation condition exhibited smooth convergence with limited divergence between curves, suggesting stable learning dynamics within the present validation design. The corresponding accuracy curves showed progressive improvement across epochs, reaching a plateau in the final training stages (Figure 1).

**Figure 2:**
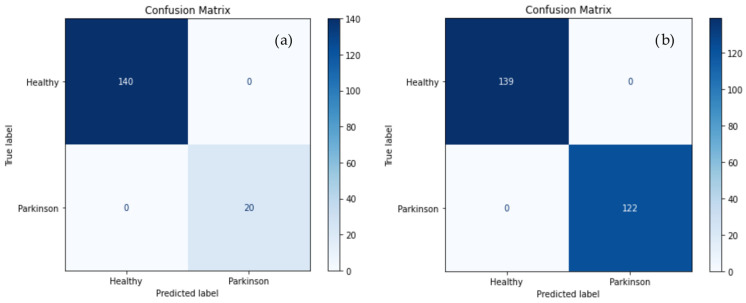
Confusion matrices for classification between healthy subjects and patients with PD: (**a**) no augmentation; (**b**) selective augmentation applied exclusively to PD sequences.

Confusion matrices obtained under selective PD augmentation showed low rates of false positives (healthy classified as PD) and false negatives (PD classified as healthy), indicating improved classification reliability relative to other configurations (Figure 2).

Additional analyses of augmentation intensity indicated that increasing PD augmentation up to a factor of ×5 using the selected time-warping parameters resulted in optimal model performance. Further increases in augmentation intensity or modifications to the time-warping standard deviation (σ) were associated with progressive performance degradation, suggesting a limited range of variability that benefits model training without degrading signal structure. These results should be interpreted within the scope of the present dataset and evaluation design.

## 4. Discussion

The present study introduces and validates a targeted data augmentation strategy grounded in the preservation of physiologically meaningful motor entropy in healthy gait and the selective modulation of pathological movement patterns. Across all experimental configurations, this entropy-aware approach consistently outperformed conventional augmentation strategies, including uniform augmentation and healthy-only augmentation, supporting the hypothesis that data augmentation in clinical AI should reflect underlying motor control principles rather than rely solely on statistical balance.

Selective augmentation of Parkinsonian gait patterns data, while preserving the natural variability of healthy movement, resulted in the highest classification accuracy (94.1%) and discriminative performance (AUC = 0.97). Sensitivity and specificity were closely balanced, indicating that the model was equally effective at identifying pathological gait patterns and correctly classifying healthy movement. From a methodological standpoint, this balance suggests greater robustness and reduced bias toward either class, particularly in proof-of-concept settings with imbalanced datasets.

The stability observed in training and validation loss curves further supports the effectiveness of the selective augmentation strategy. Unlike full-class augmentation, which showed reduced validation performance, selective PD augmentation maintained close alignment between training and validation metrics in the present evaluation design. Confusion matrix analysis confirmed low rates of both false positives and false negatives, indicating improved separation between healthy and pathological gait patterns at the segment level.

Importantly, model performance declined when the augmentation factor exceeded ×5 or when the time-warping standard deviation (σ) deviated from the selected range. This observation suggests a constrained range of variability within which synthetic perturbations remain beneficial. Excessive augmentation likely disrupts salient temporal gait features, reducing the model’s ability to generalize within the validation setting. Rather than unlimited variability being advantageous, these results indicate that augmentation effectiveness depends on remaining within physiologically plausible bounds. This concept may be interpreted as an “entropy envelope” within which pathological signal variability enhances learning without distorting meaningful temporal structure.

From a motor control perspective, this finding aligns with established entropy-based frameworks of human movement. Healthy gait is characterized by rich yet structured variability driven by adaptive neuromuscular control and continuous micro-adjustments to internal and environmental demands [24,25]. Excessively perturbing this variability risks compressing the natural entropy space and obscuring discriminative features. By contrast, Parkinsonian gait is marked by reduced dynamical complexity due to bradykinesia, rigidity, and impaired adaptability [26,27]. In this context, controlled augmentation, such as smooth time-warping, may introduce variability consistent with temporal fluctuations, thereby expanding the pathological signal space within realistic physiological limits [28,29,30,31].

This entropy-sensitive design contrasts with the widespread use of symmetric augmentation strategies reported in previous gait classification studies, which apply identical transformations indiscriminately to both classes [32,33]. While such approaches can increase dataset size and apparent balance, they often treat augmentation as a purely mathematical operation, detached from the physiological meaning of signal variability [34,35]. The present results suggest that this assumption may be suboptimal when class variability differs intrinsically, as is commonly observed in Parkinsonian locomotor patterns.

From an application-oriented perspective, preserving healthy gait variability may help maintain specificity in gait pattern classification by avoiding distortions of normal movement dynamics [36,37,38]. Conversely, selectively augmenting pathological gait signals improves sensitivity by expanding intra-class variability without overriding constrained temporal organization. However, these interpretations remain preliminary and must be validated in larger cohorts and in subject-independent evaluation designs. Accordingly, the present approach should be interpreted as phenotype-level gait pattern recognition rather than a disease-specific diagnostic framework, which would require integration with biological markers and disease-control cohorts to support claims of specificity within modern PD nosology.

Beyond PD, the principles demonstrated here may extend to other movement disorders characterized by distinct variability profiles. Conditions such as ataxia, post-stroke hemiparesis, or multiple sclerosis exhibit different patterns of motor variability and adaptation. In these contexts, augmentation strategies could be tailored to reflect disorder-specific temporal dynamics rather than applying symmetric transformations. More broadly, integrating pathophysiological knowledge into preprocessing and augmentation pipelines may improve robustness and interpretability in time-series movement analysis.

Several limitations should be acknowledged. Despite augmentation, the dataset, particularly for PD gait sequences, remains modest in size, and validation on larger, multi-center cohorts is necessary to assess generalizability. In addition, evaluation was performed at the segment level, and augmentation does not generate new independent subjects; therefore, performance metrics should be interpreted as internal comparative indicators rather than subject-independent estimates. Experimental constraints may also have influenced gait biomechanics, as different treadmill configurations were used for healthy and PD participants for safety reasons. Furthermore, clinical variables such as medication status, disease stage or axial symptom burden were not systematically controlled and may affect gait variability and temporal structure. Future studies may address this limitation. Finally, hypogravity-assisted walking should be viewed primarily as an experimental paradigm rather than a direct surrogate of everyday overground gait. By partially unloading body weight while maintaining continuous locomotion, the Motigravity platform provides a controlled and repeatable environment to examine gait timing regulation, adaptability, and motor coordination under altered loading constraints. This setting is particularly valuable for feasibility-oriented studies in which signal quality, participant safety, and standardized acquisition conditions are essential, especially in vulnerable populations. However, hypogravity locomotion remains a non-ecological condition that may modify sensory integration, balance demands, and postural control compared with natural walking. Future studies should employ a unified treadmill configuration across groups, or explicitly model treadmill type and walking speed as covariates, particularly when investigating higher-speed or more challenging locomotor tasks.

The augmentation strategy was restricted to time-warping; future work could explore adaptive or hybrid augmentation schemes guided by subject-specific variability characteristics. In addition, future benchmarking against classical machine-learning baselines under identical preprocessing and validation settings will be necessary to isolate the specific contribution of selective augmentation from model architecture effects. Studies incorporating more diverse and balanced populations will be important to assess robustness across demographic subgroups.

Finally, entropy-aware augmentation may offer opportunities to improve model interpretability [39,40,41]. Examining how learned representations evolve in response to controlled changes in signal variability could help bridge the gap between deep learning models and physiologically grounded interpretation, contributing to more transparent AI systems for movement analysis.

## 5. Conclusions

This study demonstrates that selective, entropy-aware data augmentation can enhance classification performance in Parkinsonian gait pattern analysis compared with conventional symmetric augmentation strategies. By preserving the natural motor entropy of healthy gait while selectively enriching variability within pathological signals, the proposed approach achieves a biomimetic balance consistent with known physiological characteristics of Parkinsonian locomotor impairment.

Beyond improving gait classification performance in the present experimental setting, these findings suggest that data augmentation in clinical AI may benefit from explicit grounding in physiology-based motor control principles. Treating augmentation as a physiology-aware intervention rather than a uniform technical step may improve model stability and interpretability in constrained datasets. Importantly, this work is positioned as an exploratory methodological contribution, and future studies using larger cohorts, disease-control populations, and subject-independent validation will be required to assess generalizability and specificity.

## Figures and Tables

**Table 1:** Comparative performance of the CNN–LSTM model under different data augmentation strategies. AUC: Area Under the Curve; PD: Parkinson’s disease; σ = standard deviation of the Gaussian perturbation used in time-warping.

Augmentation Strategy	Accuracy (%)	AUC	Sensitivity (%)	Specificity (%)
Selective PD (×5, fixed σ)	94.1	0.97	93.8	94.3
Full-Class Augmentation	90.2	0.93	89.5	90.8
Healthy-Only Augmentation	87.4	0.91	86.9	87.8
No Augmentation	85.6	0.89	85.2	86.0

## Data Availability

The raw data underlying the study results can be obtained upon request from the corresponding author.

## Abbreviations

The following abbreviations are used in this manuscript:

PD	Parkinson Disease
IMU	Inertial Measurement Units
CSV	Comma-Separated Values
BVH	Biovision Hierarchy
CNN	Convolutional Neural Network
LSTM	Long Short-Term Memory
ROC	Receiver Operating Characteristic
AUC	Area Under the Curve
